# Damage Assessment of Through-Cracked-Bending Laminated Glass Elements Under Low-Velocity Hard-Body Impacts

**DOI:** 10.3390/ma18194454

**Published:** 2025-09-24

**Authors:** Chiara Bedon, Nicola Cella, Riccardo Del Bello

**Affiliations:** Department of Engineering and Architecture, University of Trieste, 34127 Trieste, Italy; nicola.cella@phd.units.it (N.C.); riccardo.delbello@dia.units.it (R.D.B.)

**Keywords:** laminated glass, fracture, damage, out-of-plane bending, modal analysis

## Abstract

The post-fracture mechanical performance of laminated glass (LG) members is well-known to be challenging to assess due to the influence of multiple factors. Even more challenging and scarcely explored is the assessment of the behavior of broken LG elements as a function of the degree of damage that affects it. In this paper, the attention is given to the experimental analysis of 2-ply, small-scale, pre-fractured LG elements composed of annealed (AN) glass and characterized by two different types of interlayers, namely the polymeric Ethylene-Vinyl Acetate (EVA) or the ionoplast SentryGlas^®^ (SG) bonds. The samples—with total size of 200 mm in length by 50 mm in width—are subjected to *n* = 10 repeated hard-body impact tests, in a three-point-bending (3PB) setup, to simulate and assess a possible increase in the damage severity. To quantify and compare the behavior of the different interlayers in use, experimental modal analyses are performed both at the beginning of the impact tests (*n* = 0) and after each hard-body impact repetition (*n* = 1, …, 10), by means of roving hammer tests based on #14 different control points. The comparison of the experimental outcomes—in particular, the fundamental vibration frequency *f*_1_—gives evidence of a markedly different mechanical response from the EVA and SG interlayers. EVA samples exhibited a major reduction in terms of fundamental frequency, indicating significant propagation of damage following impact repetitions. On the other hand, SG samples appear to be less seriously affected by hard-body impacts.

## 1. Introduction

Due to the intrinsic brittleness of glass, the post-fracture performance assessment of laminated glass (LG) members requires major attention in order to ensure sufficient residual capacity and robustness in case of accidental damage [1]. In particular, it is possible to guarantee a residual mechanical capacity provided that a connection between the section components and the interlock of glass fragments is preserved [2]. In this regard, the bridging effect offered by the interlayer in retaining the glass fragments plays a crucial role in ensuring a minimum post-breakage capacity [3]. Obviously, any premature debonding, or even rupture of the interlayer can seriously compromise this bridge mechanism and the entire residual capacity of the LG element [4,5,6,7,8]. Furthermore, the quantification of the decrease in terms of load-bearing capacity for a given LG section as the severity of the damage increases remains challenging to assess [2]. Therefore, understanding these local post-fracture phenomena is of the utmost importance to improve the post-fracture design and analysis of LG elements. 

In this regard, a first important step is represented by the mechanical characterization of the interlayers in use [9,10,11,12,13]. Several literature studies have addressed the post-fracture response of glass systems, both at the material and component levels [2,14,15,16,17,18,19]. In particular, in [20], the mechanisms of energy dissipation due to the delamination and stretching of a polymeric viscoelastic interlayer between broken glass shards has been experimentally investigated based on a through-cracked-tensile (TCT) test. This approach has been employed extensively in the context of evaluating the bonding capacity of interlayers [21,22,23]. On the other hand, the influence of the fracture pattern on the residual resistance of LG specimens has been studied in [24] by imposing an idealized fracture pattern with aligned cracks, which set a lower-bound estimate of the bending capacity for panels with random fracture patterns. Regarding the dynamic response of structural glass elements, experimental approaches have been applied in [25,26,27,28,29] for both monolithic and LG damaged members in laboratory conditions. In [30,31], studies have been presented for in-service glass structures, for diagnostic purposes. The viscoelastic dynamic response of LG elements has been explored in [32,33], extending to the dynamic regime the static effective thickness concept from [34] and the associated shear bonding parameter *η* (with *η* = 0 in presence of weak bonding at the “layered limit” and *η* = 1 for the upper “monolithic limit” of a fully rigid connection [34]). Experimental modal analyses have been carried out for 1.5 m × 1.5 m LG plates in [35], based on roving hammer tests, in their pre- and post-impact stage. Given that no damage was visually detected as a consequence of the imposed impact, a limited variation in the monitored dynamic parameters was generally observed for the tested specimens. Also, no relevant variation was found in terms of damping ratio. In any case, the Polyvinyl Butyral (PVB) bonding was associated with higher energy absorption potential, compared to the Ethylene-Vinyl Acetate (EVA) interlayer [35,36]. Indeed, major feedback was obtained from the frequency change analysis of monolithic glass samples retrofitted by anti-shatter safety films, in the progressively degrading post-fracture stage [37,38].

Following these earlier research activities, an original experimental investigation is presented in this paper to capture the residual mechanical capacity and dynamic features of small-scale pre-fractured LG samples composed of annealed (AN) glass. The samples are affected by the presence of an initial crack and subjected to the progressive damage of their constituent layers. The experimental analysis is carried out by exposing the samples to preliminary destructive quasi-static bending tests. This evaluation determines the quasi-static behavior and mechanical contribution of the different interlayers in use. In particular, two commercial products are employed for the specimens: the thermoplastic Evalam Visual^®^ produced by Pujol Group (Barcelona, Spain; “EVA” in the following”) and the ionoplast SentryGlas^®^ (SG5000 type) produced by Du Pont^TM^ (Wilmington, DE, USA—“SG” in the following). The attention is given to the comparative analysis of the elastic bending stiffness *EI*, maximum force *F*, deformation capacity *f* and ductility of these through-cracked-bending (TCB) specimens. 

Similar samples are then investigated in the dynamic regime, and subjected to *n* = 10 consecutive hard-body impacts. Prior to the execution of the hard-body impact tests (*n* = 0) and following each impact (*n* = 1, …, 10), the samples are subjected to experimental modal analysis (EMA) to quantify any propagation of damage and reduction in their residual mechanical capacity. In this regard, the vibration frequency *f*_1_ of the broken LG samples is primarily monitored as a key performance indicator, given that possible changes in vibration frequency reflect modifications in the mechanical parameters of the investigated samples. The possible sensitivity of the corresponding fundamental vibration shapes to damage is also addressed.

The experimental outcomes show that the progressive reduction in mechanical capacity for the tested samples, due to the increasing damage deriving from the hard-body impact test repetitions, strongly depends on the type of interlayer in use. Specifically, the stiffer SG bonding film is associated with a rather stable mechanical performance under consecutive impacts, and also when the number of impacts *n* increases. Conversely, the EVA bonding film exhibits a marked stiffness reduction that can be perceived from the first impact (*n* = 1), and shows a further severe mechanical degradation with the subsequent impact repetitions (up to *n* = 10).

## 2. Experiments

### 2.1. Quasi-Static TCB Tests

**Figure 1:**
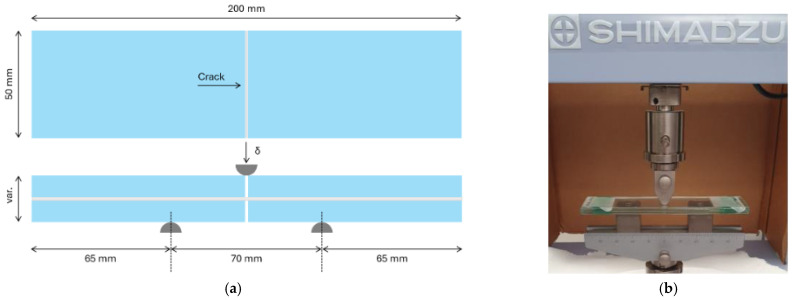
Preliminary TCB tests: (**a**) nominal geometry, with evidence of the disconnection in the mid-span section, and (**b**) test setup.

The experimental investigation consisted of a first set of preliminary TCB tests carried out on LG samples in a classical three-point-bending (3PB) setup (Figure 1). More in detail, the typical specimen consisted of a 2-ply LG element, with total length *L_tot_* = 200 mm and width fixed in *b* = 50 mm. In each LG sample, the same thickness *t_g_* was used for the two constituent AN glass layers, with *t_int_* the thickness of the bonding interlayer (Table 1). Note that Table 1 reports the nominal thickness of each glass layer (*t_g,nom_*) and its real value (*t_g,real_*), which was measured by caliper meter and found consistent with product standards [39]. The samples were specifically produced by Serex Multivitrum (Torviscosa, Italy) and assembled with an initial cut/disconnection of glass layers at the mid-span section, to reproduce a through-cracked sample, see Figure 1a. Destructive quasi-static monotonic tests were carried out with a constant loading rate of V1 = 25 mm/min (Figure 1b) to preliminarily assess and compare their out-of-plane bending performance and mechanical response. The ends of these TCB samples were simply supported by steel blocks with rounded corners (5 mm the radius), placed 65 mm apart from the ends. These blocks constrained the vertical displacement, but allowed the rotation. Thus, the distance of the end supports for the 3PB test setup resulted in *L_b_* = 70 mm.

The vertical load was applied at the mid-span section of each LG sample through a Shimadzu AGS-X universal testing machine (Shimadzu HandelsgmbH, Korneuburg, Austria), equipped with a rounded loading device (5 mm the radius), which also recorded the force–displacement response for the tested specimen with its own data acquisition system. All the tests were conducted in laboratory conditions to ensure constant ambient parameters throughout the experimental program, and each test series involved at least three specimens for each configuration, for statistical purposes, as specified in Table 1.

Based on further preliminary tensile tests carried out on virgin dog-bone EVA and SG interlayer samples (with V1 = 25 mm/min the imposed displacement rate), their modulus of elasticity was quantified in *E_int_* = 4.2 MPa for EVA and *E_int_* = 147.2 MPa for SG, respectively (see Table 1).

According to the available literature formulation for the effective thickness of LG beams [32,33,34], the expected shear bonding parameter *η* is also reported in Table 1 for the examined configurations [34]. Specifically, it is calculated under the assumption of fully intact LG samples in a simply supported setup subjected to a mid-span concentrated load [34]. The *η* parameter accurately expresses the shear coupling contribution that a given interlayer can provide to the bonded glass components. As can be seen in Table 1, the shear bonding parameter depends on the cross-section composition.

### 2.2. Hard-Body Impact Tests

**Figure 2:**
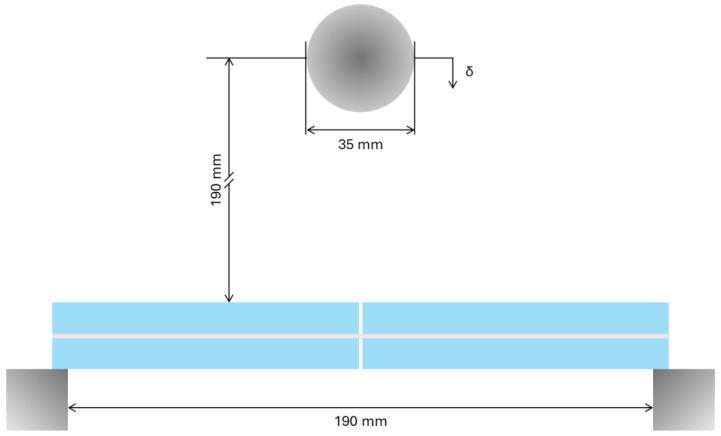
Hard-body impact test setup (out-of-scale scheme).

In the second stage of the investigation, LG specimens with features identical to those in Table 1 were tested under low-velocity hard-body impact. Each sample was positioned on two rigid blocks at the ends, with a total free span equal to *L_b_* = 190 mm. Then, a steel sphere was used for the drop ball test. The target region for impact was fixed to coincide with the mid-span section/initial crack of each LG sample. The drop height for the steel ball was fixed in *h_drop_* = 19 cm (Figure 2).

Considering that a steel ball with a diameter of 35 mm was used for the impact tests, the examined configuration corresponded to a theoretical impact energy of about *E_imp_* = 0.328 J, where

(1) $$E_{imp}=\frac{1}{2}m_{ball}v_{ball}^{2}=\frac{1}{2}m_{ball}\left(2gh_{drop}\right)$$

with *m_ball_* ≈ 0.18 kg the mass of the steel ball, *v_ball_* ≈ 1.93 m/s its velocity at the time of impact and *g* = 9.81 m/s^2^ the acceleration of gravity.

Through the experimental investigation, each LG sample was subjected to a series of *n* = 10 impacts, by keeping fixed the setup shown in Figure 2. All the impact tests were carried out under laboratory conditions and alternated with roving hammer tests for EMA purposes (see Section 2.3).

### 2.3. Pre- and Post-Impact Roving Hammer Tests and Modal Analysis

Through the impact experiments, careful attention was paid to the mechanical assessment of the tested LG specimens to quantify any potential propagation of damage due to the hard-body setup.

To this aim, experimental modal tests were performed on each LG specimen, both at the beginning of the impact series (i.e., undamaged TCB specimens, *n* = 0) and after each hard-body impact repetition (up to *n* = 10 for the final configuration).

**Figure 3:**
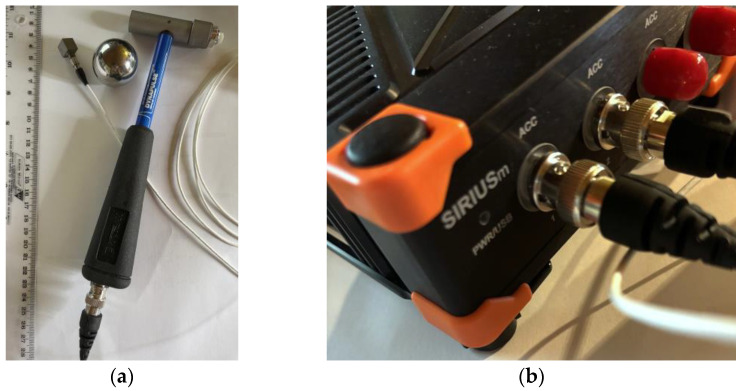
Instruments in use for roving hammer tests and experimental modal analysis: (**a**) DYTRAN^®^ modal hammer and (**b**) SIRIUS^®^ Mini acquisition system.

According to Figure 3, the modal analysis was carried out with the support of dedicated equipment distributed by DEWESoft^®^ (Trbovlje, Slovenia). An instrumented modal hammer manufactured by DYTRAN^®^ Instruments Inc. (Los Angeles, CA, USA) with 440 N as the force range was used to impose impacts (see Figure 3a). Vibrations were measured by means of a single-axis accelerometer (DYTRAN^®^), with 50 g the acceleration range and 100 mV/g its sensitivity, based on IEPE (Integrated Electronics Piezo-Electric) technology. The limited dimensions (10.2 mm for each side) and total weight (4.3 grams) of the miniature-sized cube accelerometer ensured the absence of possible interferences with the LG specimens during the dynamic tests. The sampling rate for data acquisition was fixed at 5000 Hz. Experimental data were collected through a USB-powered, 4-channel SIRIUS^®^ Mini acquisition system by DEWESoft^®^ (Figure 3b) and post-processed with the support of the dedicated DEWESoft-X^®^ software (v. 2025.2) and the dedicated plugin for modal analysis [40]. 

**Figure 4:**
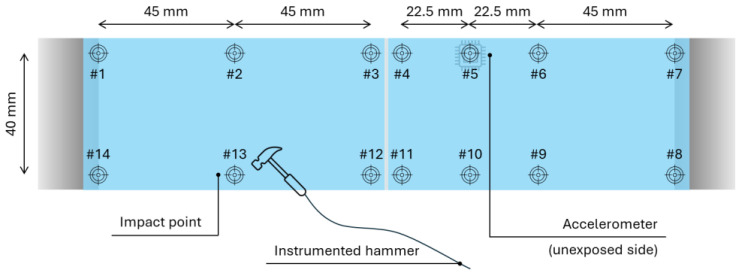
Scheme of the roving hammer tests and modal analysis setup (top view).

More in detail, each LG sample was instrumented with the accelerometer in use before the beginning of the series of impact tests (*n* = 0). The sensor was rigidly bonded to the glass at control point #5, close to the mid-span section (Figure 4). A small adhesive tape was also used to keep the cable in position during vibrations. Roving hammer tests for the modal analysis were carried out by considering a total of #14 control points on each sample (Figure 4). The typical roving hammer test was performed at the initial stage and after each hard-body impact repetition, for a total of *n* = 10 test repetitions. Moreover, the experimental records at each one of the #14 control points were collected and elaborated as the average from 3 hammer hits. Accordingly, the post-processing stage for each LG sample was performed by considering a total of (#14 × 3) × (*n* = 10) = 420 experimental records (420 for the EVA bonded sample and 420 for the SG bonded sample).

## 3. Experimental Results

### 3.1. Quasi-Static TCB Tests

As expected, the preliminary analysis of LG specimens in the TCB configuration revealed a significantly different mechanical response and bonding contribution from the EVA and SG interlayers in use, as well as from the different interlock of glass layers on the compression side of the 3PB setup. In this regard, it is important to note that the limited number of performed tests was chosen to support the dynamic experiments only. However, additional test repetitions and possible influencing parameters (aging, displacement rate, etc.) should be taken into account to derive generalized conclusions.

**Figure 5:**
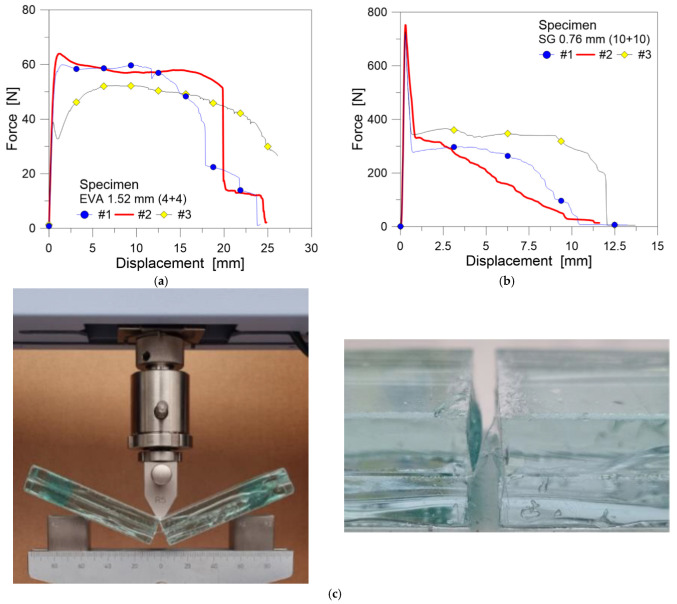
Preliminary destructive quasi-static TCB tests: force–displacement curves for (**a**) EVA and (**b**) SG bonded samples, with (**c**) details of the local deformation of the interlayer (during and after test).

A typical example of force–displacement results is proposed in Figure 5, as obtained from the three different samples described in Table 1. In general, a rather good consistency of test results was observed for each series. Notably, the maximum force *F* achieved by the EVA or SG bonded samples in the elastic stage differs by a factor of about 10. However, there are also major differences in terms of maximum recorded displacement at failure, which implicitly derive from the bridging contribution of the interlayers at the mid-span section.

For the EVA bonded samples, see Figure 5a; the response exhibited a rather stiff elastic stage with a linear increase in the sustained force *F* up to ≈60 N for an average deflection of about *f* ≈ 0.65 mm. This stage was followed by a plastic branch with a relatively stable increase in force and deflection. This branch is followed by a sudden drop in force, which corresponds to the final rupture and tearing of the interlayer at the mid-span section. Overall, the EVA bonded samples showed a rather ductile behavior after the elastic stage. In average terms, the ultimate deflection was quantified in about *f* ≈ 21 mm. Large residual deformations were observed in the interlayer after the conclusion of the test, as shown in Figure 5c for one of the samples. These experiments ended with the complete rupture of the film. At this stage, no marked debonding was observed around the mid-span section by visual inspection. 

Moving to the SG bonded specimens in Figure 5b, a mostly different response can be observed. The force–displacement response is characterized by an initial sharp increase in force up to *F* ≈ 750 N (*f* ≈ 0.32 mm the corresponding deflection), followed by a pronounced and fast force reduction down to about *F* ≈ 350 N, which derives from the contact mechanism of the glass layers on top. Afterwards, a sudden loss of capacity can be noted, compared to the EVA bonded samples. The average failure displacement was quantified in about *f* ≈ 10 mm and corresponded to the brittle rupture and tearing of the interlayer. In terms of deformation capacity, a marked reduction (in the order of −52%) can be noted for the SG specimens compared to the EVA bonded samples.

**Figure 6:**
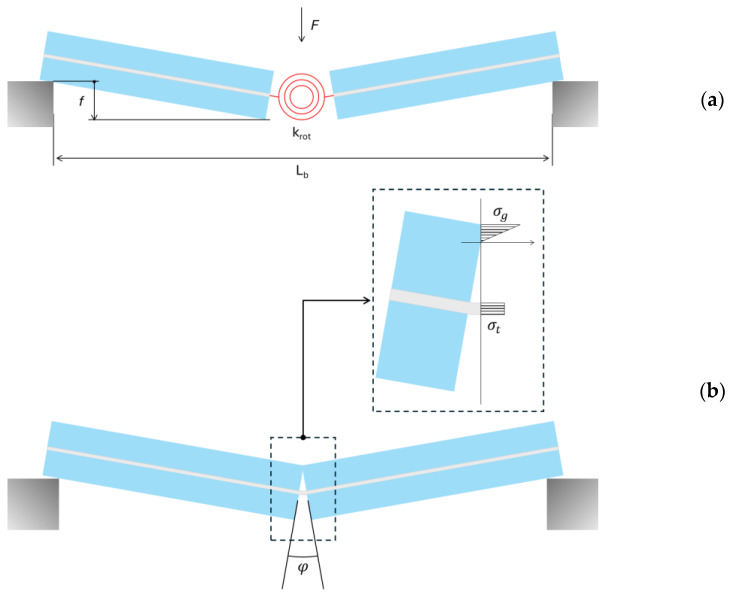
Mechanical modeling of TCB specimens: (**a**) equivalent lumped rotational spring and (**b**) stress distribution for the specimen in out-of-plane bending.

From a mechanical point of view, each LG sample can be considered in the form of a simplified model in which the mid-span crack and the interlayer bridging mechanism correspond to a lumped rotational stiffness *K_rot_* for the plastic hinge that deforms between two rigid LG arms (Figure 6a). The bridge contribution of the interlayer, see Figure 6b, can be exploited as long as the film does not fail in tension. In parallel, the compressive strength governs the resisting contribution deriving from the glass components in contact.

As an alternative to Figure 6, a distributed, equivalent out-of-plane bending stiffness *EI* can be assigned to the LG specimen to capture its mechanical response in the presence of a mid-span crack.

At the end of the linear elastic branch of Figure 6a,b, the measured mid-span deflection of each sample is in fact given by

(2) $$f=\frac{FL^{3}}{48EI}$$

with:

(3) $$M=\frac{FL}{4}$$

and:

(4) $$EI=\frac{FL^{3}}{48f}$$

The corresponding rotation for the plastic hinge at the mid-span section can be quantified in

(5) $$\varphi =\frac{4f}{L}$$

The associated rotational spring stiffness *K_rot_* is given by

(6) $$K_{rot}=\frac{M}{\varphi }$$

and results—for the setup of Figure 1—in about 2.08 × 10^5^ Nmm/rad and 5.46 × 10^6^ Nmm/rad for EVA and SG samples, respectively. In parallel, Equation (4) results in an average equivalent quasi-static elastic bending stiffness equal to *EI* = 4.56 × 10^5^ Nmm^2^ and 1.64 × 10^7^ Nmm^2^ for the EVA and SG samples. These initial stiffness values derive partly from the interlayer in use and partly from the contact mechanism for the glass layers on the compressive side (Figure 6b).

### 3.2. Hard-Body Impact Tests

The effects of each hard-body impact test were monitored by means of visual inspection of the tested specimens. Attention was given both to the propagation of possible cracks and to the monitoring and quantification of possible shards detached from the LG sample, as well as to the detection of any major damage phenomena in the interlayer.

**Figure 7:**
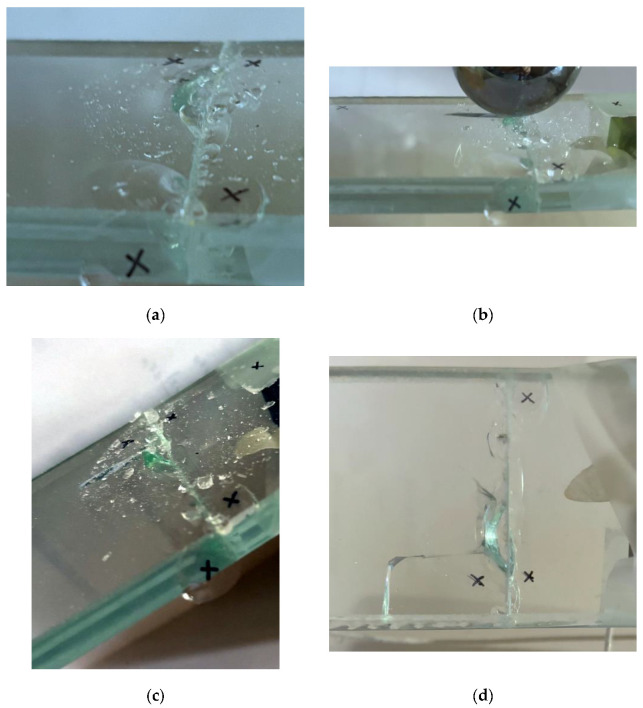
Progressive damage analysis in the EVA bonded specimen subjected to repeated hard-body impacts (out-of-scale photos): (**a**) minor fracture on the impact surface after *n* = 2; (**b**) transversal crack in the top glass layer after *n* = 3; (**c**) impact surface after *n* = 4; (**d**) final configuration, after *n* = 10 (bottom view).

Overall, the EVA bonded specimen appeared more severely affected by the imposed impacts and suffered for the propagation of secondary cracks in the region of the mid-span section (top glass layer). In any case, no relevant fragments detached from the sample, and the total mass of the specimen remained largely unchanged throughout the execution of the *n* impact repetitions. Figure 7 shows a selection of detailed views of the impact region.

**Figure 8:**
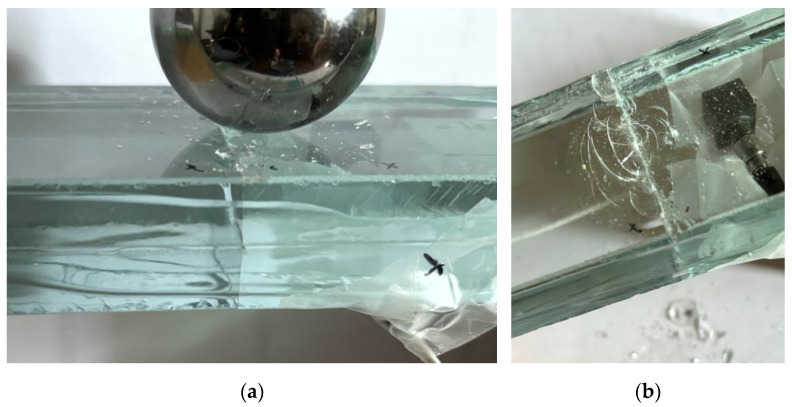
Progressive damage analysis in the SG bonded specimen subjected to repeated hard-body impacts (out-of-scale photos): (**a**) detail after *n* = 1; (**b**) final configuration, after *n* = 10.

In the case of the SG bonded sample, a rather different mechanism was visually observed during hard-body impact repetitions. Specifically, no secondary cracks were noted to propagate in the glass components. Minor or negligible shards were only detected on the impact surface (Figure 8), especially towards the end of the series of impacts. As in the previous case, the total mass of the sample remained largely unchanged throughout the tests. In both cases, no damage phenomena were visually detected in the EVA or SG interlayers.

### 3.3. Roving Hammer Tests and Modal Analysis

During the roving hammer tests, the maximum force in the hammer was generally measured in the order of ≈40–50 N, for both EVA and SG samples. Accordingly, the maximum acceleration peaks acquired in the sensor in control point #5 were measured in peaks of about ≈250 m/s^2^. 

The post-processing of Frequency Response Function (FRF) signals was primarily focused on the fundamental vibration mode, which is the most sensitive for the examined LG samples and hard-body impact setup. Considering, for example, an “intact/continuous” LG member with the same features as the tested specimens (excluding the preliminary TCB crack at mid-span), the fundamental shapes of the lower modes for this simply supported sandwich member coincide with the classical flexural shape of a beam. The TCB crack at mid-span and the viscoelastic response of the interlayers should also be properly assessed. Through the analysis of the experimental results, careful consideration was paid to the quantification of the dynamic modulus *E_int_* of EVA and SG interlayers, as it depends—among others—on the displacement rate and load duration. For the purpose of the present study, two additional intact LG samples were hence tested by roving hammer and investigated with classical modal analysis steps to extrapolate the dynamic *E_int_* value. This procedure resulted in *E_int_* = 9.7 MPa for EVA and *E_int_* = 198 MPa for SG samples.

In this regard, it is important to note that the shear bonding parameter *η* recalled in Table 1 [32,33,34] would result (due to the different setup and mechanical properties) in about *η* = 0.323 for EVA and *η* = 0.829 for SG, respectively.

**Figure 9:**
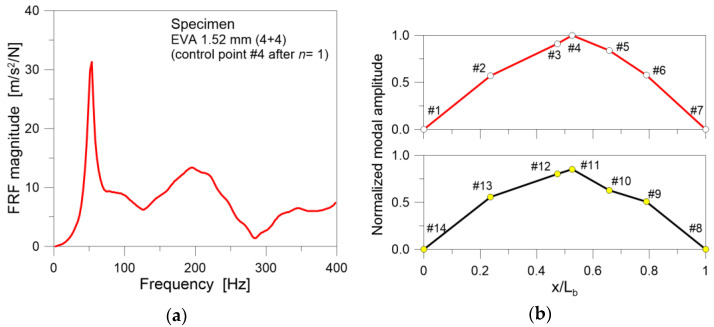
Example of modal analysis results for the EVA sample after *n* = 1: (**a**) FRF signal and (**b**) normalized fundamental vibration shape.

The modal analysis of TCB specimens, as expected, gave evidence of different quantitative variations and hard-body impact effects for the EVA or SG bonded samples, for several reasons. Figure 9 shows an example of FRF signals (Figure 9a) and the fundamental, flexural vibration shape (Figure 9b). 

For a more detailed and quantitative analysis of experimental results, the attention was hence focused on the fundamental vibration frequency *f*_1_, which represents a preliminary but important monitoring parameter that can be used to quantify the equivalent bending stiffness *EI* of the tested samples and any progression of damage. Considering that the dynamic tests were carried out at an average room temperature of 28 °C, the FRF signal in Figure 9a for the EVA bonded sample reminds the results reported in [32,33] for PVB bonded LG specimens. Specifically, it was shown that, due to the viscoelastic response of the interlayer in use, the vibration modes higher than the first one are generally less pronounced and fairly identifiable from experimental modal analysis at operational temperatures higher than 20 °C. 

Under these assumptions and considering that the elastic mechanical characterization of the viscoelastic interlayers in the dynamic regime is known, the quantification of the mid-span crack/damage effects was analyzed based on *f*_1_. In particular, assuming *f*_1,*n*=0_ as the vibration frequency of the TCB sample at the initial stage of impact tests (*n* = 0), it can be expected that

(7) $$f_{1,n\;}<f_{1,n=0}\;\left(n=1,\ldots ,10\right)$$

However, the analytical challenge of post-cracked LG configurations, as in the present study, derives from the joint analysis of (i) the intrinsic LG features (and, in particular, the viscoelastic behavior of EVA and SG), (ii) the presence of the initial TCB crack at mid-span and (iii) the possible progression of damage through the experimental series.

For a simply supported, continuous beam-like member with monolithic glass section (*t* its total thickness), the fundamental vibration frequency is

(8) $$f_{1\;}=k_{1}^{2}\;\sqrt{\frac{EI}{\rho A}}$$

with *E* the modulus of elasticity, *I* the second moment of area, *A* the cross-section, *ρ* the material density and *k*_1_ = π/*L_b_* the wavelength. Considering the examined test results (i.e., Figure 8 and Figure 9), and extending the concept of Equation (8) to a LG member with equivalent thickness *t*, the basic assumption of the present study is that *E*, *ρ*, *A* did not change during the experimental run. Accordingly, Equation (8) suggests that any possible reduction in the fundamental frequency *f*_1_ derives from a decrease in the equivalent *I*, which implicitly accounts for the mechanical features of the interlayers in use.

The intrinsic challenge of the examined LG samples, which is represented by the initial TCB crack at the mid-pan section, requires further considerations, as this crack represents a first major source of discontinuity (Figure 6). In the case of a single major crack, as in the present investigation, the cracked frequency *f_1_*_,cr_ of an equivalent monolithic glass beam (*t* its thickness) can usually be estimated according to the approach previously applied to glass in [29], that is,

(9) $$f_{1,cr}=f(f_{1},size,depth,crack\;position,etc.)$$

Assuming that the crack is located at mid-span section and has propagation *a*= 0 ÷ *t* through the thickness *t* of glass, Equation (9) takes the form [29]

(10) $$f_{1,cr}=f_{1}\sqrt{\frac{1+2\eta }{1+4\eta +\left(\pi^{4}/24\right)\eta }}$$

with *f*_1_ given by Equation (8), and

(11) $$\eta =\frac{t}{L_{b}}m\left(\frac{a}{t}\right)$$

where the function *m*(*a*/*t*) is given by

(12) $$m\left(\frac{a}{t}\right)=2{\left(\frac{a/t}{1-a/t}\right)}^{2}\left[5.93-19.69\left(\frac{a}{t}\right)+37.14{\left(\frac{a}{t}\right)}^{2}-35.84{\left(\frac{a}{t}\right)}^{3}+13.12{\left(\frac{a}{t}\right)}^{4}\right]$$

However, the above analytical model is unable to account for a sandwich LG section, as in the present case, and the mid-span mechanism which is typical of LG members in the post-cracked stage (Figure 6). In this regard, the introduction of the equivalent thickness concept for LG members (i.e., an equivalent bending stiffness *EI* for the composite section) could effectively capture the strain rate sensitivity of EVA and SG, as well as the progressive degradation effects of the examined LG samples. With this strategy, any minor damage in the impact region resulting from local phenomena (i.e., in the adhesion of interlayer film) could be implicitly taken into account. In addition to the viscoelastic response of EVA and SG interlayers, their possible local debonding—which is another diagnostic aspect—would be implicitly accounted by means of a frequency change analysis.

The study in [32,33], in this regard, proposed an analytical formulation for the dynamic equivalent thickness of LG members, which accounts for the stiffness sensitivity of typical interlayers to strain rate and temperature. Assuming that the fundamental frequency is still given by Equation (8), the corresponding equivalent bending stiffness EI* for a 2-ply LG member as in the present study can be calculated as

(13) $$EI=EI^{*}=Eb\left(\frac{t_{g}^{3}}{6}+\frac{t_{g}{\left(t_{g}+t_{int}\right)}^{2}g^{*}}{1+2g^{*}}\right)$$

with

(14) $$g^{*}=\frac{G_{int}\;L_{b}^{2}}{E\;t_{g}t_{int}\;k_{1}^{2}}$$

where *G_int_* is the shear modulus of EVA or SG, corresponding to the examined loading configuration.

**Figure 10:**
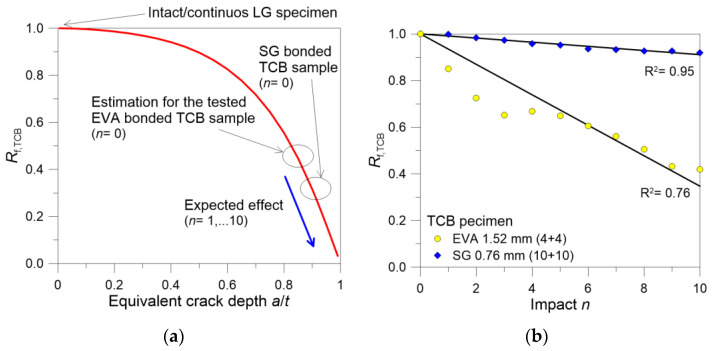
Fundamental frequency reduction *R*_f,TCB_ for the examined LG specimens: (**a**) analytical estimates based on Equations (9)–(12) and (**b**) experimental results for the TCB samples subjected to repeated hard-body impacts.

In support of the discussion of experimental results, Table 2 compares the ratio *R*_f,TCB_ calculated from the change in the fundamental vibration frequency of the tested samples in the initial TCB stage (*n* = 0) and the final stage (*n* = 10) towards the theoretical “intact/continuous” LG layout (i.e., without the TCB crack). These last values are calculated analytically by means of Equation (13), given that the geometrical features and stiffness of the interlayers are known. Moreover, Figure 10a proposes the analytical trend of the fundamental vibration frequency for the tested TCB samples, based on Equations (9)–(12).

As shown Table 2, the TCB crack at mid-span induces a severe reduction in the equivalent bending stiffness *EI*, which is captured by a marked frequency decrease and by the corresponding *R*_f,TCB_ ratio. For the presently examined setup, it can be noted that the thicker the glass layer, the more severe the effect of the TCB discontinuity. In addition, Table 2 reveals a different progression of damage when comparing *R*_f,TCB_ for *n* = 0 and *n* = 10. The same effect is also analytically emphasized by Figure 10a. Based on Equations (9)–(12), moreover, the equivalent normalized depth *a*/*t* of the initial TCB crack is analytically calculated in Table 2. This results in *a*/*t* = 0.85 for the tested EVA samples at *n* = 0 and increases to 0.945 after *n* = 10. It is interesting to note that, for the SG bonded sample, the calculated value at *n* = 0 is still in the order of 0.857 and slightly increases to 0.87 after *n* = 10. 

Concerning the quantification of repeated hard-body impacts in the intermediate steps (*n* = 1, …, 10), Figure 1b shows the experimental variation in the fundamental frequency for the examined LG samples (in terms of *R*_f,TCB_ ratio) as a function of the imposed impact *n*. The figure also reports the correlation coefficient R^2^ for the linear fit of these experimental trends. As shown, the EVA bonded sample suffers severe degradation, which is in the order of about −15% after *n* = 1, and progressively increases down to −60% at the end of the series of hard-body impacts (*n* = 10). The trend of experimental data is poorly aligned with the linear fit; this is due to the occurrence of some fracture events/adjustments for the 4 mm thick glass layers of the tested sample (see also Figure 7).

According to Figure 10b, the SG bonded sample looks less seriously affected by the repetitions of hard-body impacts. However, it still shows signs of damage propagation in the interlayer. This is quantified by a frequency reduction which approaches −8% at the end of the experimental series (*n* = 10). Considering that the 10 mm thick glass layers did not manifest major cracks (see Figure 8), such a frequency degradation can be justified by the progressive damage of the SG interlayer in the region of the mid-span section.

**Figure 11:**
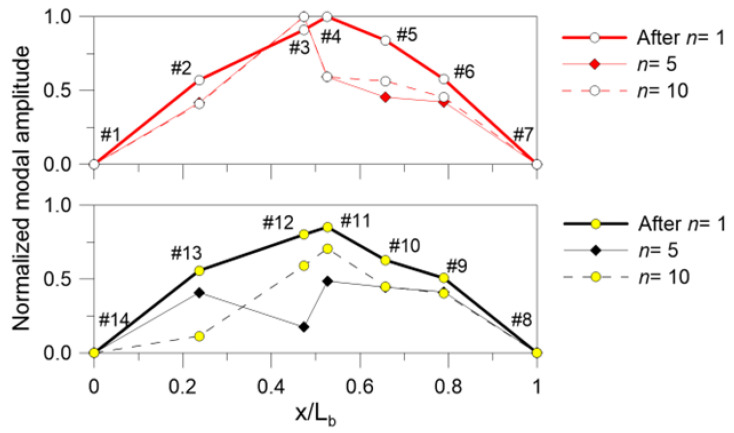
Variation in the normalized fundamental modal shape for the EVA sample, as a function of *n* (selection).

Finally, Figure 11 shows the variation in the experimentally derived fundamental modal shape for the EVA bonded sample, which is—similarly to the frequency change—a diagnostic tool for damage detection [31]. The damage severity can be clearly perceived from the selected normalized displacements through the series of repeated impact tests, which was not observed for the SG sample. Certainly, these local observations in terms of modal shape sensitivity to damage are limited by the number and features of the tested specimens. As such, generalized observations cannot be derived for diagnostic purposes. In addition, the presented results emphasize the importance and potential of similar dynamic performance indicators and parameters to support the post-fracture assessment of glass structures.

## 4. Conclusions

Quantifying damage assessment and its effects on the post-fracture mechanical performance of laminated glass (LG) members is rather challenging due to the influence of multiple factors. This paper focuses on the experimental analysis of small-scale through-cracked-bending (TCB) laminated glass (LG) samples in a traditional three-point-bending (3PB) setup. These samples were subjected to repeated low-velocity hard-body impact tests. A total of *n* = 10 impacts were considered, with *E_imp_* = 0.328 J the associated impact energy.

The analysis of experimental results was primarily focused on the fundamental vibration frequency of the 2-ply specimens and its possible sensitivity to a series of impact repetitions and progressive damage (if any). To this aim, the specimens were assembled with a width of 50 mm, a total length of 200 mm, a glass thickness of 4 mm or 10 mm, and a preliminary mid-span fracture for both the constituent glass layers. 

Two different interlayers were considered for the tested LG samples: the Evalam Visual^®^ and the SG5000^®^ commercial products. The analysis of the experimental results gave evidence of marked differences in their dynamic response and reaction to progressive damage due to hard-body impact. In particular, the comparative analysis showed major degradation effects for the EVA bonded samples rather than SG samples. The fundamental frequency reduction was in fact quantified in −60% in the first case (after *n* = 10), and only in −8% (after *n* = 10) for the SG sample. Accordingly, the associated normalized vibration shape was also analyzed. The experimental observations further confirmed some important shape modifications for the EVA sample due to the imposed repeated impacts, whilst a more consistent bending shape was generally observed for the SG sample. Overall, the limited number of experimental results does not allow for general conclusions to be drawn, as many other influencing parameters should be taken into account. However, the study proposed important outcomes that could guide future studies.

## Figures and Tables

**Table 1:** List of specimens for the preliminary quasi-static TCB tests. * = average experimental results from quasi-static tensile tests on dog-bone interlayer samples (V1 = 25 mm/min).

Series	*t_g,nom_* [mm]	*t_g,real_* [mm]	*t_int_* [mm]	Interlayer	*E_int_* [MPa]	*η*	Displacement Rate [mm/min]	n. of Specimens
EVA bond	4	3.814	1.52	EVA	4.2 *	0.171	25	3
SG bond	10	9.766	0.76	SG	147.2 *	0.784	25	3

**Table 2:** Frequency analysis for the EVA and SG bonded samples. Key: * = based on dynamic modal analysis; ** = analytical frequency based on Equation (13).

	*E*_int_ MPa	*f*_1_ Hz			*R*_f,TCB_		a/t	
Series	Dynamic *	Intact/ Continuous	TCB (*n* = 0)	TCB (*n* = 10)	*n* = 0	*n* = 10	*n* = 0	*n* = 10
EVA bond	9.7	142.67 **	60.91	25.52	0.43	0.18	0.851	0.945
SG bond	198	285.29 **	73.93	67.98	0.25	0.23	0.857	0.870

## Data Availability

The original contributions presented in this study are included in the article. Further inquiries can be directed to the corresponding author.
